# Institutional Questions

**Published:** 1899-09-30

**Authors:** 


### INSTITUTIONAL QUESTIONS.

### Lockers and Food.

"A Hospital Visitor" writes: I am much surprised to find that at some of the chief metropolitan and provincial hospitals it is still the custom for patients to be permitted to supply part of their own diet, such as butter, sugar, and tea, and that such food is kept by the side of the bed, in the "locker," together with a heterogenous collection of personal properties. Surely this is a very insanitary arrangement, and incompatible with the principles of modern medical science?

Tradition and custom are hard things to get over, and evil habits die hard. The practice of keeping food of any kind, but especially such an article as butter, in a ward at all is entirely opposed to every principle of hygiene. Nevertheless, the plan is one which has existed for generations, and too often hospital committees have a way of thinking that what "was good enough twenty years ago is good enough now." If the medical men attached to those hospitals where this indefensible custom prevails—and we are glad to think that there are comparatively few—would make a practice of looking into the patients' lockers as they go round the wards and observing for themselves the internal arrangements of these bedside lockers, we fancy strongly-worded protests would soon find their way to the boards of management of such institutions, on the score that they constitute a positive danger to health. A specimen of butter taken from a ward locker should form the subject of an interesting discourse upon bacteriology! It is a curious fact that as a question of economy statistics show the cost per bed to be relatively higher at those hospitals which demand the most from their in-patients in the matter of supplies, a fact which leaves no possible excuse for the continuance of an antiquated and objectionable practice.

### Rounded Corners.

"Kirkcudbright" asks: Is it desirable while building a new hospital—quite a small one—to have the corners rounded after the approved fashion followed in more important institutions? Does this entail much extra expense?

It certainly is a great advantage to dispense with angles, and all dust-catching corners should be avoided as far as possible. If you discuss the matter with your architect you will find that the extra expense involved is not very serious, and the committee would do wisely in making their hospital as up-to-date as may be in these details of construction.

### Ladies' Committees.

"A Correspondent" writes: We have a small hospital in this neighbourhood which has hitherto been managed by a committee of men. Some ladies have recently shown considerable interest in its welfare and the patients, and it is proposed to form a "Ladies' Committee." Will you give me your views on this matter! Is such a course likely to produce friction between the "ladies" and the nursing staff, who have a theoretical objection to this innovation?

It depends pretty much upon the constitution of the Ladies' Committee, and the limits set to their authority whether the plan will work well in practice or not. It is a better plan to elect a few ladies upon your general committee than to form a new body with independent powers, which, brought suddenly into existence, is more than likely to follow the example of the proverbial new broom, and sweep cleaner than may be convenient. If you have a competent matron or nurse in charge it is certainly better to leave the management of domestic details to her, under the supervision of the general committee, and from what you mention of existing conditions we should judge it to be a mistake to take any action likely to disturb present harmony. The presence of a few ladies on your general committee could, however, only be productive of good.

